# Enrichment of Inflammatory IL-17 and TNF-α Secreting CD4^+^ T Cells within Colorectal Tumors despite the Presence of Elevated CD39^+^ T Regulatory Cells and Increased Expression of the Immune Checkpoint Molecule, PD-1

**DOI:** 10.3389/fonc.2016.00050

**Published:** 2016-03-07

**Authors:** Margaret R. Dunne, Ciara Ryan, Bláthnaid Nolan, Miriam Tosetto, Robert Geraghty, Des C. Winter, P. Ronan O’Connell, John M. Hyland, Glen A. Doherty, Kieran Sheahan, Elizabeth J. Ryan, Jean M. Fletcher

**Affiliations:** ^1^School of Biochemistry and Immunology, Trinity Biomedical Sciences Institute, Trinity College Dublin, Dublin, Ireland; ^2^School of Medicine, Trinity Biomedical Sciences Institute, Trinity College Dublin, Dublin, Ireland; ^3^Department of Pathology, St. Vincent’s University Hospital, Dublin, Ireland; ^4^Centre for Colorectal Disease, Education and Research Centre, St. Vincent’s University Hospital, Dublin, Ireland; ^5^School of Medicine, University College Dublin, Dublin, Ireland

**Keywords:** colorectal cancer, T cells, regulatory T cells, Th17 cells, PD-1, immunophenotyping, immunotherapy

## Abstract

T cell infiltration into colorectal tumors has been shown to correlate with improved patient outcomes. However, more detailed information on the makeup and relationships between the infiltrating T cell subsets is lacking. We therefore correlated the extent of immune infiltration into colorectal tumors with the frequencies of various T cell subsets. We prospectively recruited 22 patients at the time of surgical resection for colorectal cancer. The Klintrup–Mäkinen (KM) score was used to estimate the extent of immune infiltration into colorectal tumors. The frequencies of CD4 and CD8 T cells that produced cytokines or expressed the inhibitory molecule programed cell death 1 (PD-1) were determined by flow cytometry in colorectal tumor and matched uninvolved colonic tissue. In addition, the frequency of CD4 regulatory T cell (Treg) subsets was determined. An increased frequency of CD4 T cells producing IL-17 (Th17 cells) was observed in colorectal tumor tissue compared with adjacent uninvolved tissue. These Th17 cells mostly coproduced TNF-α, but not IFN-γ. IL-17 expression correlated positively with TNF-α and IL-10. Increased expression of the immune checkpoint molecule PD-1 was found in colorectal tumors compared with adjacent uninvolved tissue. There was a negative correlation between expression of PD-1 and IFN-γ, but not IL-17, for both CD4^+^ and CD8^+^ T cells. CD4^+^CD25^+^CD127^lo^ and CD4^+^CD25^+^CD127^lo^FoxP3^+^CD39^+^ Treg cells were enriched in colorectal tumors. A positive correlation between KM score and percentage CD4^+^CD25^+^CD127^lo^ Treg cells was observed in tumors, suggesting that increased immune infiltration is associated with an increased proportion of Treg cells. In addition, there was a negative correlation between the frequency of CD4^+^CD25^+^CD127^lo^ Treg cells and the expression of IFN-γ and IL-2, but not IL-17, in tumors. Taken together, these data suggest that both PD-1 expressing T cells and Treg cells within the tumor may have a suppressive effect on T cells secreting IFN-γ, IL-2, or TNF-α, but not Th17 cells.

## Introduction

There is accumulating evidence indicating that the number, type, and location of tumor infiltrating lymphocytes has prognostic value in colorectal cancer (CRC), where robust T cell infiltration correlates with improved outcome (1, 2). These data have led to the development of the immunoscore, derived from measurement of memory (CD45RO) CD3 and CD8 T cell infiltration into the tumor center, and invasive margin. Significantly, the immunoscore correlated positively with improved outcome regardless of stage and is being validated in large scale studies (3).

However, tumor infiltrating T cells contain a number of different functional subtypes, which can have either pro- or antitumor effects. Cytotoxic IFN-γ-producing CD8 T cells play a key role in the antitumor response. The role of CD4 T cells, which can be divided into Th1, Th2, Th17, and regulatory T cell (Treg) cell subsets, is more complex. Th1 cells, which produce IFN-γ and provide help to CD8 T cells, are considered to have an antitumor role (4). However, the role of Th17 cells in tumor immunity remains controversial. In the context of autoimmunity Th17 cells are pro-inflammatory and pathogenic. Although studies in murine cancer models indicate an antitumor role for Th17 cells (5–7), there is contrasting evidence from other murine and human studies, suggesting that Th17 cells promote angiogenesis and drive tumor development (4, 8, 9). IL-17 promotes angiogenesis by inducing VEGF production by tumor cells (9) and can mediate resistance to anti-VEGF therapy in murine models (10). Furthermore, the tumor microenvironment promotes the recruitment and expansion of human Th17 cells (11). Importantly, patients with low expression of Th17-related genes exhibited prolonged disease-free survival (4). Thus on balance, the data from human studies appear to favor a model, where Th17 cells promote angiogenesis and tumor development.

Regulatory T cell cells play a crucial regulatory role in maintaining tolerance and preventing autoimmunity. However, in the context of cancer, the general consensus is that Treg cells inhibit antitumor responses and contribute to the immunosuppressive microenvironment. However, it is possible that Treg cells could play a dual role by initially dampening protumor inflammation, but later acting to inhibit antitumor effector cells in the established tumor. In CRC, Treg cells have been shown to be enriched in the tumor (12); however, the role of these Treg cells remains controversial (13). In contrast to findings in other cancer settings, high levels of CRC tumor infiltrating Treg cells were associated with early stage disease and improved prognosis (14, 15). Other studies, however, did not find a positive correlation between good prognosis and Treg cell infiltration (16, 17). The question of how tumor infiltrating Treg cells regulate local effector T cells within the tumor microenvironment remains to be determined.

The introduction of cancer immunotherapies, including those targeting CTLA-4 and programed cell death 1 (PD-1)/PD-L1, has highlighted the therapeutic relevance of understanding the regulation of local tumor immunity (18, 19). However, the success of immunotherapy is variable between patients and likely to be dependent on the presence of the relevant target; for example, the success of targeting the PD-1/PD-L1 axis was limited to patients with tumors expressing PD-L1 and with CD8 T cell infiltration (20), and so far these drugs have demonstrated poor clinical efficacy in CRC (21). Here, we report a detailed analysis of tumor infiltrating Treg cells and PD-1^+^ lymphocytes in parallel with documenting the cytokine producing potential of effector cells within the same tumor. Interestingly, we found that despite being highly infiltrated by potentially immunosuppressive CD39^+^ Treg cells, colorectal tumors contain significant numbers of pro-inflammatory IL-17 and TNF-α secreting T cells. These effector T cells are likely to be a key component of the local tumor-sustaining inflammatory environment that is increasingly being recognized as a characteristic of solid tumors (22).

## Materials and Methods

### Patient Samples

Colorectal tumor and adjacent histologically normal tissue samples (at least 10 cm from tumor site) were obtained from 22 patients undergoing surgical resection at St. Vincent’s University Hospital, Dublin. Patient demographics and clinical details are reported in Table 1. Informed consent was obtained from all participants. Ethical approval for this study was granted by the St Vincent’s Healthcare Group Ethics and Medical Research Committee, and research was conducted according to the Helsinki guidelines.

**Table 1:** **Patient characteristics**.

*N*	22
Male/female	16/6
Age (years), median (range)	69, 47–93
TMN stage	Stage I (2), stage II (9), stage III (9), stage IV (2)
Lymphovascular invasion	Present (11), absent (11)
Perineural infiltration	Present (6), absent (16)
Extramural venous invasion	Present (12), absent (10)
Mismatch repair loss	0/22
KM score	0 (2), 1 (16), 2 (4)
Tumor budding	Present (10), absent (12)
Tumor differentiation	Moderate (17), well (5)
Tumor size (cm), median (range)	4.8, 1.2–9.5
Tumor location	Sigmoid (4), caecum (4), ascending (6), transverse (1), descending (3), rectal (4)
Surgery	Curative intent (21), Hartman’s procedure (1)
Adjuvant chemotherapy recommended	Yes (10), no (12)

### Histopathological Analysis

The samples from surgical specimens were fixed in 10% buffered formalin solution and embedded in paraffin, and 5 mm sections were stained with H&E. We evaluated peritumoural inflammatory reaction from H&E slides according to the Klintrup–Mäkinen (KM) criteria, where 0 denoted no recruitment of inflammatory cells; 1 denoted mild and patchy increase of inflammatory cells; 2 denoted a band-like infiltrate at the invasive margin with some evidence of destruction of cancer cell islets; and 3 denoted a very prominent inflammatory reaction with frequent destruction of cancer cells. These scores were then classified into low grade (0–1) and high grade (2–3) (23, 24). All cases were also evaluated for the absence or presence of Crohn’s-like reaction (CLR).

### Cell Isolation

Tissue samples were collected and transported in Hank’s Balanced Saline Solution, calcium- and magnesium-free (HBSS, Sigma-Aldrich), supplemented with 1% FBS (Sigma) and PENSTREP, Gentamycin, and Fungizone (Labtech) (Life Technologies). Tissue fragments were washed thoroughly, chopped, digested in 125 U/ml collagenase Type IV-S, and then sterile filtered (Sigma-Aldrich) in HBSS. Tissue was incubated at 37°C with agitation, for no longer than 30 min. Cells were then filtered through a 70-nm nylon mesh (BD Biosciences), washed in warm HBSS, and counted. Cells were either stained immediately for Treg cell markers and T cell memory markers or incubated overnight in complete RPMI medium (supplemented with 10% FBS and PENSTREP) prior to restimulation with PMA (10 ng/ml) and ionomycin (1 μg/ml) in the presence of brefeldin A (5 μg/ml) (all Sigma-Aldrich) for 3.5 h.

### Flow Cytometry Staining

For analysis of PD-1, cells were washed in serum-free PBS and stained with a fixable viability dye, eFluor506, CD4-PerCPCy5.5, PD1-PECY7, CD8-APCeFluor780 (eBioscience), incubated for 15 min at room temperature in the dark, and then washed in PBS buffer containing 1% FBS and sodium azide. Cells were stained for Treg cell markers using a FoxP3 staining buffer set (eBioscience) and accompanying protocol. Treg cell markers included CD39-FITC, FoxP3-PE, CD73-PerCPeFluor710, CD25-PECY7, CTLA-4-APC, CD127-APCeFluor780, Ki67-eFluor450 (eBioscience), and CD4-V500 (BD Biosciences). An intracellular staining kit (Fix and Perm kit, Invitrogen) was used to analyze cytokine production after restimulation with PMA/ionomycin. Cells were stained with IL-17A-AlexaFluor488, IL-10-PE, TNF-α-PerCPCy5.5, CD45RA-PECY7, CD8-APCeFluor780, FoxP3-eFluor450 (all eBioSciences), CD45 AlexaFluor700 (BioLegend), IFN-γ-APC, CD3-V500, and IL-2-PE-CF594 (BD Biosciences). Due to PMA/ionomycin-mediated reduction in CD4 expression, CD4^+^ T cells were identified as CD3^+^CD8^−^ T cells for cytokine analysis. Cells were acquired on a BD LSRFortessa flow cytometer and analyzed using FlowJo software (Flowjo LLC).

### Statistical Analysis

Analysis of statistical differences in T cell population frequency and cytokine production between paired uninvolved tissue and tumor samples was performed using Prism GraphPad version 6. All data were assumed to be non-parametric and were analyzed using the Wilcoxon matched pairs test. Pearson’s correlation coefficient (*r*) was calculated using the Statistical Package for the Social Sciences (SPSS) version 18.0, IBM.

## Results

### Varied Grades of Immune Cell Infiltration into Colorectal Tumor Tissue

We prospectively recruited 22 patients at the time of surgical resection for CRC. Details of the patient cohort are shown in Table 1. All patients were treatment-naive at the time of surgery. Mismatch repair (MMR) status for all cases was determined by immunohistochemistry for the MMR proteins, MLH1, PMS2, MSH2, and MSH6. None of the tumors included in our analyses displayed microsatellite instability (MSI).

**Figure 1:**
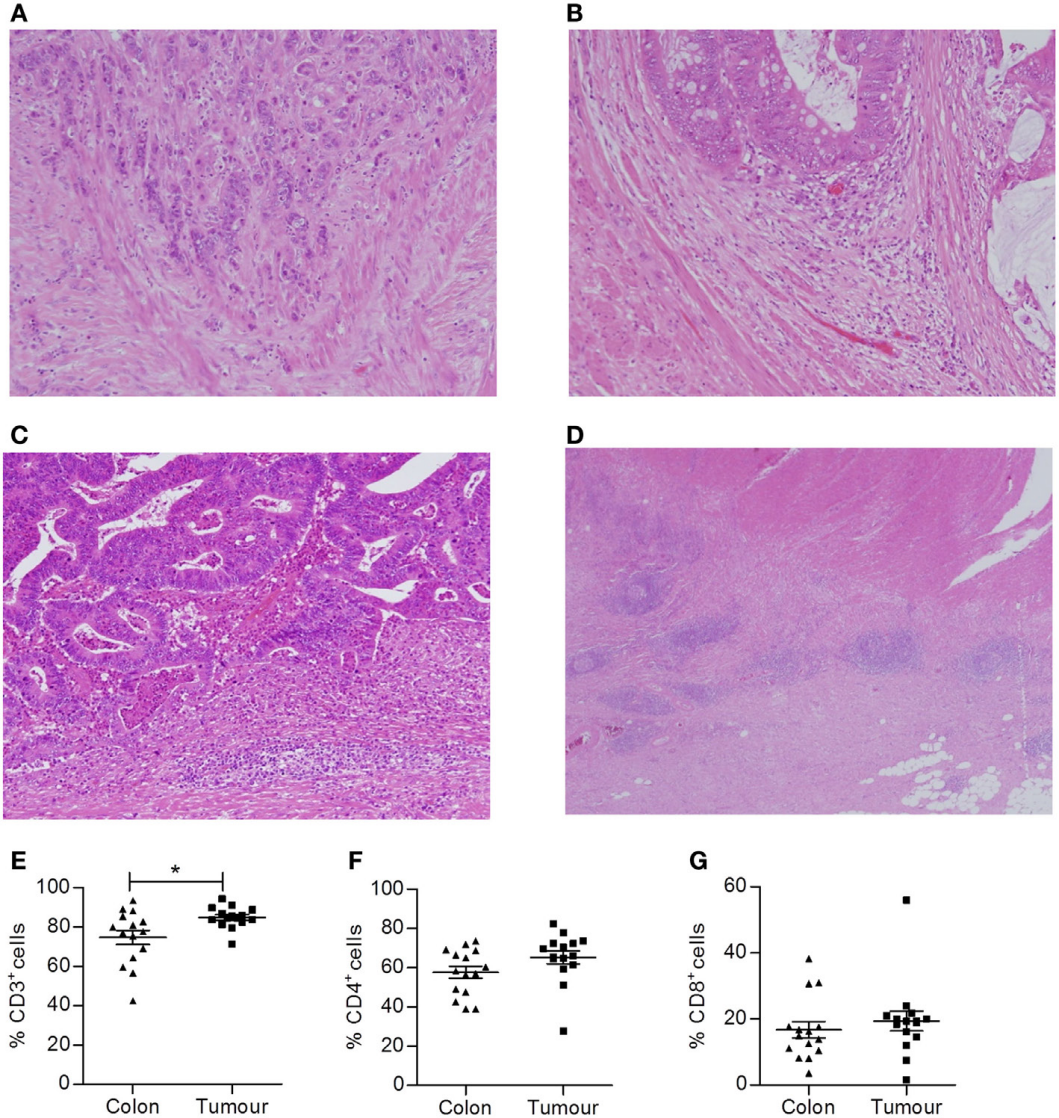
**Colorectal tumors are highly infiltrated with T cells**. Representative H&E slides of colorectal tumors with varying KM scores. **(A)** KM score 0, **(B)** KM score 1, **(C)** KM score 2, and **(D)** Crohn’s-like reaction. Single cell suspensions were also obtained by enzymatic digestion from surgically excised normal adjacent colonic tissue and colorectal tumors. The frequency of CD3^+^ **(E)**, CD4^+^ (CD3^+^CD8^−^) **(F)**, and CD8^+^ **(G)** T cells within lymphocytes was determined by flow cytometry. *p < 0.05 as determined by the Wilcoxon signed rank test.

In this study, we used the KM score to estimate the extent of immune infiltration into colorectal tumors. After surgical resection, colorectal tumor tissue samples were paraffin embedded, sectioned, and H&E stained. Representative examples of KM scores 1–3 are shown in Figures 1A–C. Of the 22 cases examined, only *n* = 2 had a KM score of 0, indicating that the majority of MSS tumors included in this study were infiltrated by immune cells. We also documented the absence or presence of a CLR in all patient samples. An example of Crohn’s-like staining is shown in Figure 1D. Of the 22 tumors examined, 15 displayed evidence of a CLR, again indicating a significant degree of immune cell infiltration in the tumors of this cohort.

In addition, histologically normal adjacent tissue and tumor tissue were finely chopped and enzymatically digested to obtain single cell suspensions that were analyzed by flow cytometry to assess the phenotype of tumor infiltrating T cells. Cells were stained with antibodies specific for CD45 to identify tumor infiltrating leukocytes and panels of antibodies specific for T cell markers to identify specific subsets of T lymphocytes. Compared to uninvolved adjacent tissue, colorectal tumor tissue had an increased frequency of total T cells (*p* < 0.05) (CD3^+^ lymphocytes, Figure 1E). However, both the uninvolved normal tissue and colorectal tumor tissue were infiltrated by CD4^+^ and CD8^+^ T cells, with a CD4:CD8 ratio of approximately 2:1 (Figures 1F,G). The vast majority of T cells infiltrating tumors were found to be of the effector memory type (data not shown). However, within the immune infiltrate, the ratio of CD4^+^:CD8^+^ T cells did not differ from uninvolved adjacent tissue.

### Increased Frequency of CD4^+^IL-17^+^ (Th17) Cells Infiltrating Colorectal Tumors

**Figure 2:**
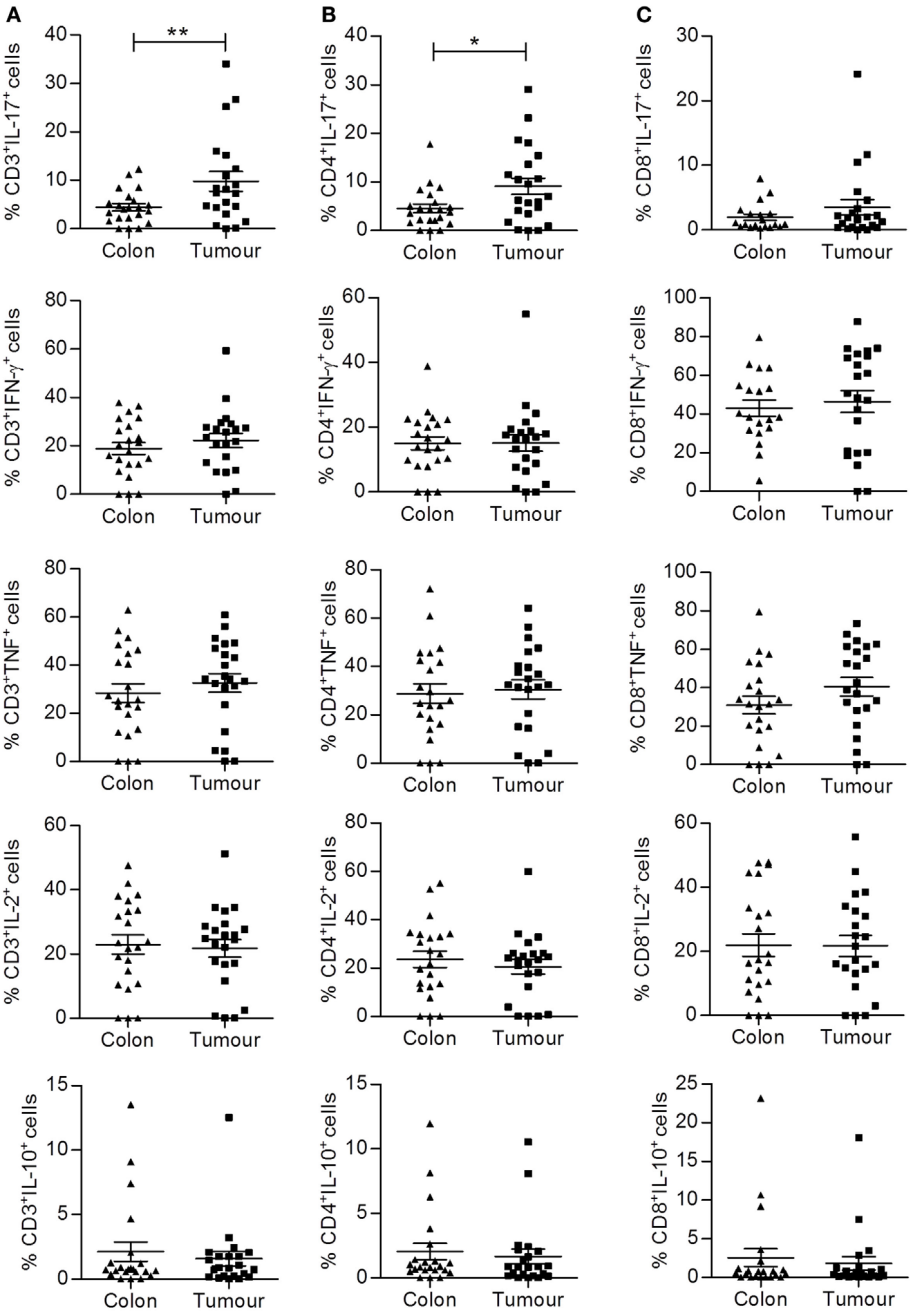
**Increased frequency of CD4^+^IL-17^+^ (Th17) cells infiltrating colorectal tumor tissue compared to normal adjacent tissue**. Tumor tissue and adjacent uninvolved colonic tissue were enzymatically digested, and the resulting single cell suspension was stimulated with PMA and ionomycin. Cells were stained with antibodies specific for CD3 and CD8, permeabilized, and stained for intracellular expression of cytokines IFN-γ, IL-17, TNF, IL-2, and IL-10. The frequency of cytokine expressing total CD3^+^ **(A)**, CD4^+^ (CD3^+^CD8^−^) **(B)**, or CD8^+^ **(C)** T cells is shown for uninvolved colon and colorectal tumor tissue. *p < 0.05, **p < 0.01 as determined by Wilcoxon signed rank test.

We then aimed to further dissect the phenotype and function of these colorectal tumor infiltrating lymphocytes by analyzing cytokine production by individual T cell subsets using intracellular staining and flow cytometry. Single cell suspensions from normal adjacent tissue and colorectal tumor tissue were prepared from fresh surgical explants and stimulated for 3.5 h with PMA and ionomycin in the presence of brefeldin A. Cells were then stained with antibodies specific for T cell markers, permeabilized, and incubated with antibodies specific for the following cytokines: IFN-γ, IL-17A, TNF-α, IL-2, and IL-10. No differences in the frequency of total CD3^+^, CD4^+^, or CD8^+^ T cells producing IFN-γ, TNF-α, IL-2, or IL-10 were observed between uninvolved intestinal tissue and tumor tissue (Figures 2A–C). There was a significant increase in the frequency of total CD3^+^ (*p* < 0.01) and CD4^+^ (*p* < 0.05) T cells producing IL-17 (Figures 2A,B), indicating that the local microenvironment of colorectal tumors can selectively enhance Th17 cell polarization or recruitment.

### Tumor Infiltrating Th17 Cells Coproduce TNF-α, but Not IFN-γ

Having found significantly elevated frequencies of Th17 cells within the pool of lymphocytes recruited to colorectal tumors, we wished to further characterize this T cell subset. Th17 cells have been observed to exhibit plasticity of function and often coproduce IFN-γ, particularly at sites of inflammation. Cells that produce both IL-17 and IFN-γ are thought to be more pathogenic in inflammatory disease, but conversely would be more effective antitumor effectors. Therefore, we investigated if the tumor infiltrating Th17 cells also produced other cytokines, notably IFN-γ or TNF-α, as this would provide crucial information regarding their potential function.

**Figure 3:**
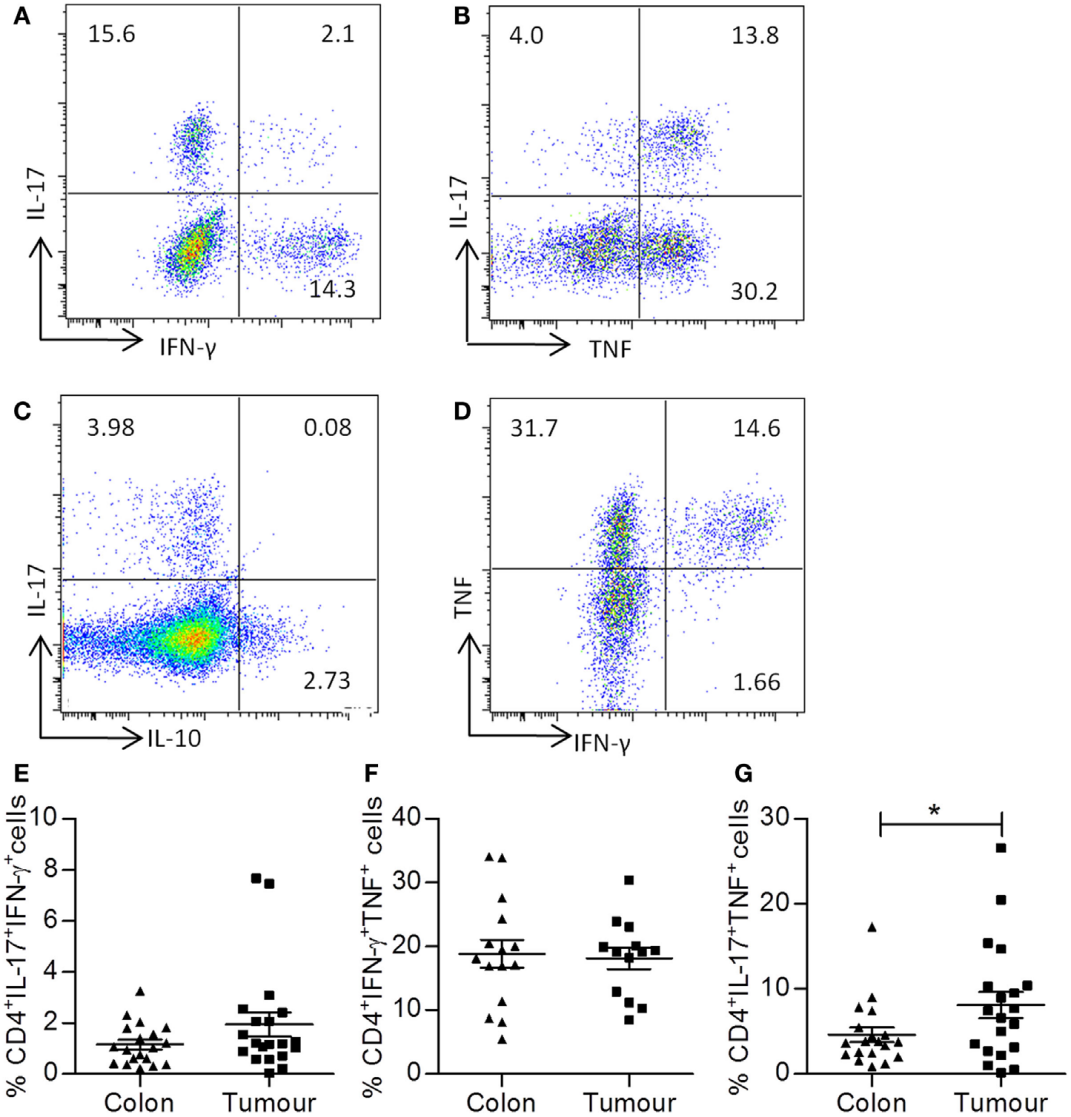
**Th17 cells infiltrating colorectal tumors coexpress TNF-α, but not IFN-γ**. Single cell suspensions that were obtained from tumor tissue and adjacent uninvolved colonic tissue were stimulated with PMA and ionomycin. Cells were stained with antibodies specific for CD3 and CD8, permeabilized, and stained for intracellular expression of the cytokines TNF-α, IFN-γ, and IL-17. Representative dot plots were gated on CD4^+^ (CD3^+^CD8^−^) T cells and show expression of IL-17 versus IFN-γ **(A)**, IL-17 versus TNF-α **(B)**, IL-17 versus IL-10 **(C)**, or TNF versus IFN-γ **(D)**. The frequencies of CD4^+^IL-17^+^IFN-γ^+^ **(E)**, CD4^+^TNF^+^IFN-γ **(F)**, or CD4^+^IL-17^+^TNF-α^+^ **(G)** T cells are shown for uninvolved colon and colorectal tumor tissue. *p < 0.05 as determined by the Wilcoxon signed rank test.

Representative dot plots shown in Figure 3 were gated on tumor infiltrating CD4^+^ T cells and show IL-17 versus IFN-γ (Figure 3A), IL-17 versus TNF-α (Figure 3B), IL-17 versus IL-10 (Figure 3C), and TNF versus IFN-γ (Figure 3D) staining. There was no significant increase in the frequency of CD4^+^ T cells that coproduced IL-17 and IFN-γ (Figure 3E) or TNF and IFN-γ (Figure 3F). In addition, there was virtually no coexpression of IL-17 and IL-10 observed in tumor samples (Figure 3C). However, a significant increase in the frequency of IL-17^+^TNF-α^+^ CD4^+^ T cells was observed in tumor tissue relative to adjacent uninvolved intestine (*p* < 0.05) (Figure 3G). These data suggest that IL-17^+^TNF-α^+^CD4^+^ T cells may play a role in shaping the local immune microenvironment of colorectal tumors.

Consistent with the above data, a significant positive correlation between the frequencies of CD3^+^IL-17^+^ T cells and CD3^+^TNF-α^+^ T cells was observed (*p* < 0.05) (Table 2). In addition, a correlation between expression of IL-17 and IL-10 within CD3^+^ T cells was found (*p* < 0.01) (Table 2); however, this did not appear due to coexpression of these cytokines. However, IFN-γ production by T cells correlated with the production of both TNF-α (*p* < 0.01) and IL-2 (*p* < 0.01) (Table 2). These data suggest that there are few IFN-γ/IL-17 secreting T cells within colorectal tumors, with the majority of Th17 cells coproducing TNF-α. Furthermore, IFN-γ secreting T cells, when present, also secrete IL-2 in addition to TNF-α. Further understanding of the factors that contribute to these alternative cytokine profiles within the tumor infiltrating T cell pool is required.

**Table 2:** **Pearson correlation of IL-17 or IFN-γ production by tumor infiltrating CD3^+^ T lymphocytes versus other cytokines (*n* = 20)**.

	Pearson correlation versus IL-17^+^ lymphocytes
CD3^+^ IFN-γ	0.241
CD3^+^ TNF-α	0.519^a^
CD3^+^ IL-2	0.164
CD3^+^ IL-10	0.665^b^

	**Pearson correlation versus IFN-γ^+^ lymphocytes**

CD3^+^ TNF-α	0.602^b^
CD3^+^ IL-2	0.747^b^
CD3^+^ IL-10	0.163

*^a^Correlation is significant at the 0.05 level (two-tailed)*.

*^b^Correlation is significant at the 0.01 level (two-tailed)*.

### Increased Expression of PD-1 on T Cells within Colorectal Tumors

**Figure 4:**
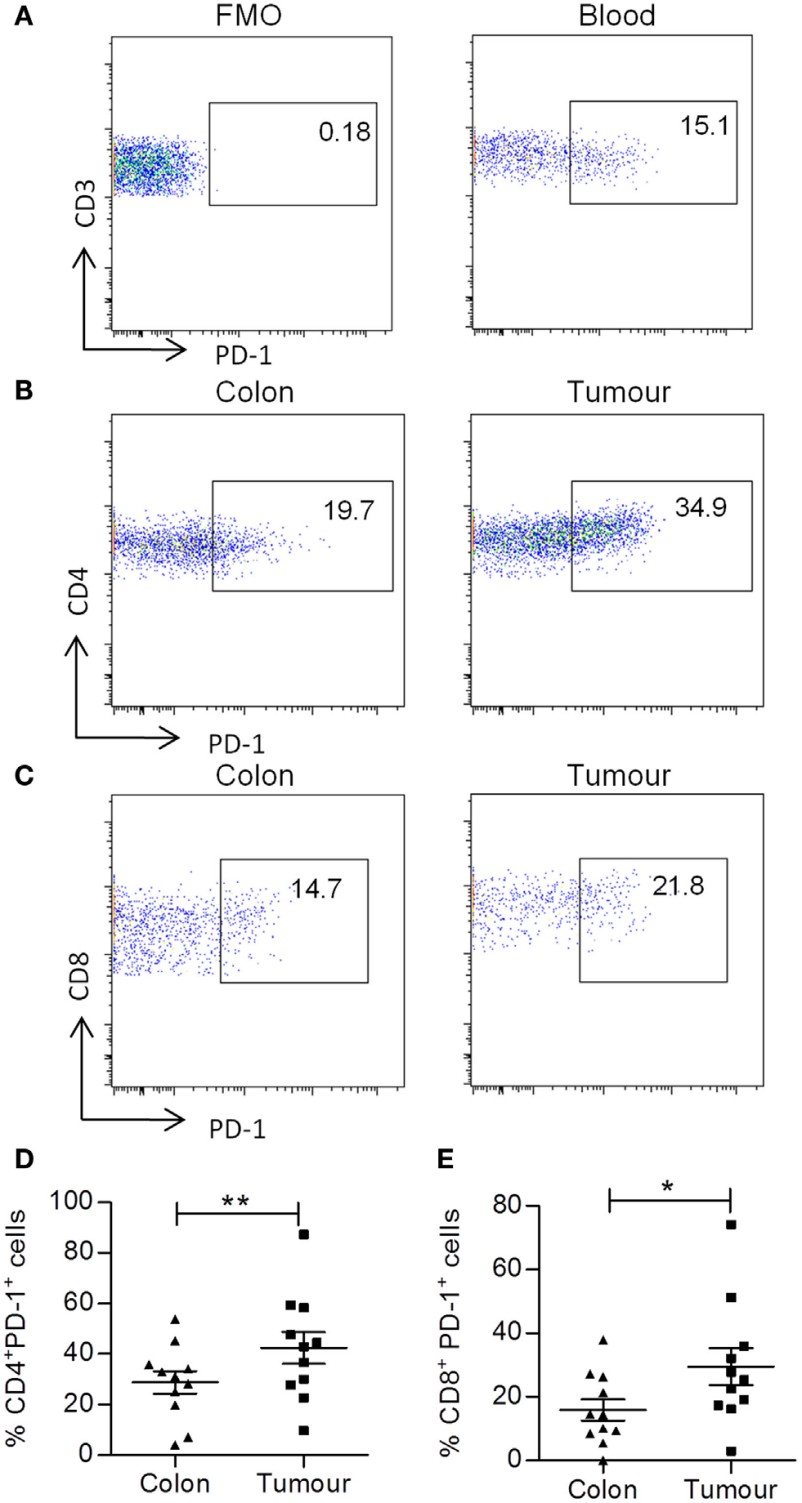
**Increased frequency of PD-1 expression on tumor infiltrating CD4^+^ and CD8^+^ T cells**. Tumor tissue and adjacent uninvolved colonic tissue were digested and stained with antibodies specific for CD3, CD4, CD8, and PD-1. Representative FMO control **(A)** and PD-1 dot plots of PD-1 staining on CD3^+^ T cells in blood **(B)**, PD-1 staining on CD4^+^ **(B)** and CD8^+^ **(C)** T cells in uninvolved colon or tumor samples are shown. The graphs show the frequency of PD-1^+^ CD4^+^ **(D)** and CD8^+^ **(E)** T cells. *p < 0.05, **p < 0.01 as determined by the Wilcoxon signed rank test.

Cytokine production is tightly regulated within human tissues by many mechanisms including cell–cell contact via the immune checkpoint, PD-1. Therefore, we investigated the expression of PD-1 on both CD4^+^ and CD8^+^ T cells in adjacent normal tissue and colorectal tumors by flow cytometry. Representative dot plots show staining controls in peripheral blood (Figures 4A,B) and PD-1 expression by colorectal tumor infiltrating CD4^+^ T cells (Figure 4B) or CD8^+^ T cells (Figure 4C). There was a significantly increased expression of PD-1 on both CD4^+^ (*p* < 0.01) (Figure 4D) and CD8^+^ (*p* < 0.05) (Figure 4E) T cells within colorectal tumor tissue relative to adjacent uninvolved tissue. Interestingly, we found that there was a negative correlation between the expression of PD-1 and IFN-γ for both CD4^+^ and CD8^+^ T cells (*p* < 0.01) (Table 3). The expression of PD-1 on total CD3^+^ T cells also correlated negatively with CD4^+^IL-2^+^ (*p* < 0.05), CD8^+^IL-2^+^ (*p* < 0.01), CD4^+^IFN-γ^+^TNF-α^+^ (*p* < 0.01), and CD8^+^IFN-γ^+^TNF^+^ (*p* < 0.05) T cells (Table 3). In contrast, there was no correlation between the expression of PD-1 and IL-17, TNF-α, or IL-10 production (Table 3). These data suggest that the increased expression of PD-1 by colorectal tumor infiltrating lymphocytes may have a suppressive effect on both CD4^+^ and CD8^+^ effector T cells secreting IFN-γ, IL-2, or TNF-α, but not IL-17 within colorectal tumors.

**Table 3:** **Pearson correlation of CD3^+^ T cell cytokine production with tumor infiltrating PD1^+^ T cells (*n* = 11)**.

CD3^+^ cytokine producing cells	Pearson correlation versus CD4^+^PD1^+^ T cells	Pearson correlation versus CD8^+^PD1^+^ T cells
IFN-γ	−0.843^b^	−0.952^b^
IL-17	−0.402	−0.426
TNF-α	−0.632	−0.492
IL-10	−0.288	−0.149
IL-2	−0.642^a^	−0.829^b^
IFN-γ+TNF-α	−0.744^b^	−0.626^a^
IFN-γ+IL-17	−0.298	−0.330
TNF-α+IL-17	−0.409	−0.368

*^a^Correlation is significant at the 0.05 level (two-tailed)*.

*^b^Correlation is significant at the 0.01 level (two-tailed)*.

### Increased Frequency of CD4^+^CD25^+^CD127^lo^ and CD4^+^CD39^+^CD25^+^CD127^lo^FOXP3^+^ Treg Cells in Colorectal Tumor Tissue

**Figure 5:**
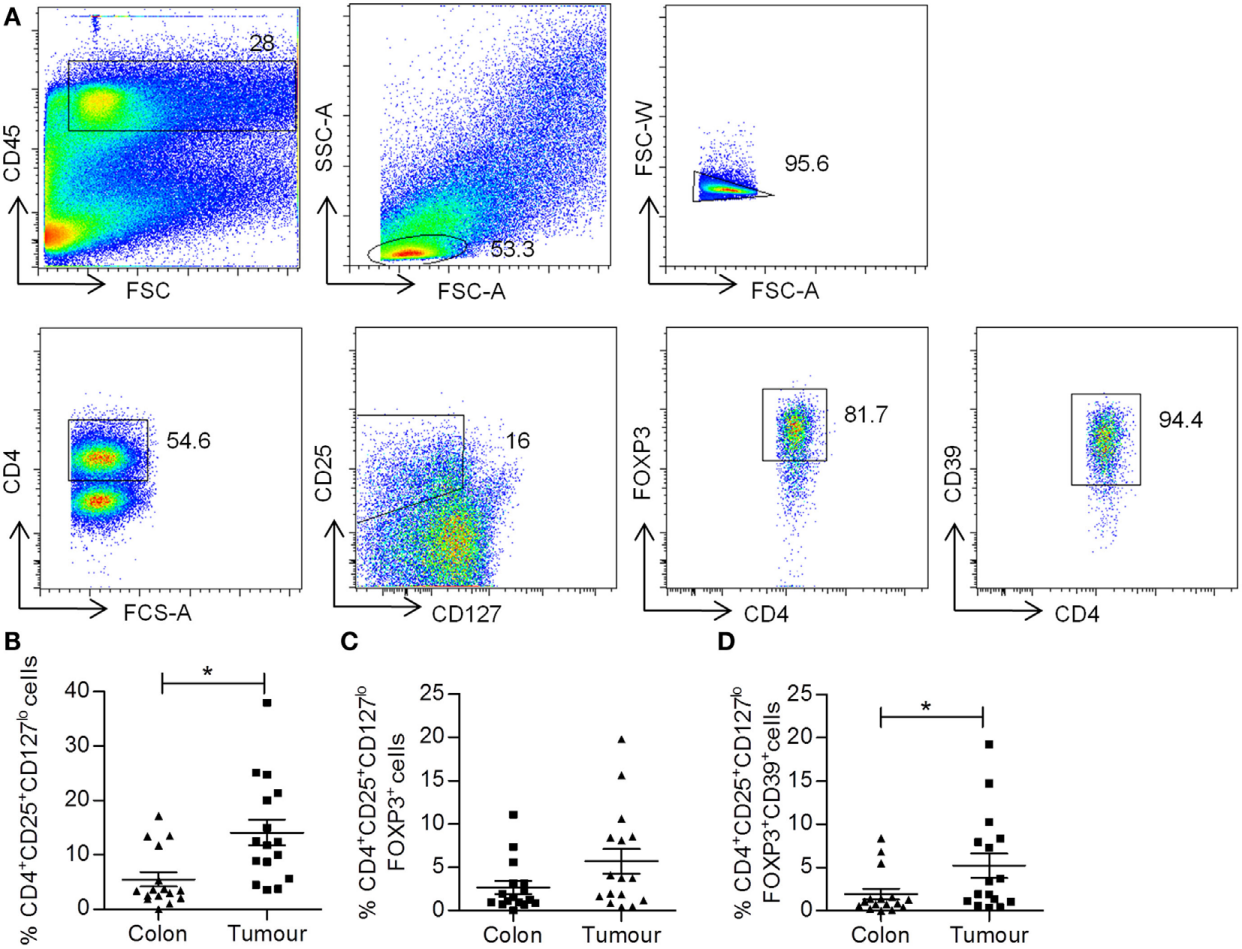
**Increased frequency of CD4^+^CD25^+^CD127^lo^ and CD4^+^CD25^+^CD127^lo^ FoxP3^+^CD39^+^ Treg cells in colorectal tumor tissue**. Tumor tissue and adjacent uninvolved colonic tissue were enzymatically digested to form a single cell suspension, which were stained with antibodies specific for CD45, CD4, CD25, CD127, FOXP3, and CD39, and were analyzed by flow cytometry to identify Treg cell populations. The sequential gating strategy used to identify Treg cell populations is shown in **(A)**. The graphs show the frequency of CD4^+^CD25^+^CD127^lo^ **(B)**, CD4^+^CD25^+^CD127^lo^FOXP3^+^ **(C)**, and CD4^+^CD25^+^CD127^lo^FOXP3^+^CD39^+^ **(D)** Treg cells. *p < 0.05, as determined by the Wilcoxon signed rank test.

Regulatory T cell cells suppress effector T cells and are thought to contribute to the immunosuppressive microenvironment of tumors. Single cell suspensions obtained from both colorectal tumors and adjacent uninvolved tissue were stained for markers of Treg cells, including CD4, CD25, CD127, FOXP3, and CD39, and were analyzed by flow cytometry. The gating strategy for the identification of Treg cell populations is shown in Figure 5A. Lymphocytes were gated on the basis of CD45^+^ and forward scatter versus side scatter, and then CD4^+^ T cells were gated. A population of CD4^+^CD25^+^CD127^lo^ Treg cells were identified, and then these were further gated on their expression of FOXP3 to identify CD4^+^CD25^+^CD127^lo^FOXP3^+^ Treg cells. The subset of Treg cells expressing CD39 was then identified (Figure 5A). An increased frequency of CD4^+^CD25^+^CD127^lo^ Treg cells was identified in tumor tissue relative to adjacent colon (*p* < 0.05) (Figure 5B), while there was no significant difference in the frequency of CD4^+^CD25^+^CD127^lo^FOXP3^+^ Treg cells (Figure 5C). However, the frequency of CD39^+^ Treg cells was significantly increased in tumor tissue relative to uninvolved tissue (*p* < 0.05) (Figure 5D). A positive correlation between KM score and the percentage CD4^+^CD25^+^CD127^lo^ Treg cells was observed in tumors (Table 4), suggesting that increased immune infiltration is associated with an increased frequency of Treg cells within the infiltrate. There was a negative correlation between the frequency of CD4^+^CD25^+^CD127^lo^ Treg cells and the expression of IFN-γ and IL-2 in tumors (*p* < 0.05) (Table 4), suggesting that Treg cells may exert a suppressive effect on effector T cell cytokines.

**Table 4:** **Pearson correlation of CD4^+^CD25^+^CD127^+^ T cells and infiltrating cytokine producing cells in colorectal tumors (*n* = 14)**.

	Pearson Correlation versus CD4^+^CD25^+^CD127^+^ T cells
KM score	0.518^a^
IL-17	−0.198
TNF-α	−0.512
IFN-γ	−0.574^a^
IL-2	−0.598^a^
IL-10	0.139

*^a^Correlation is significant at the 0.05 level (two-tailed)*.

## Discussion

In this study, we investigated the phenotype of tumor infiltrating T cells by flow cytometry in a cohort of patients with MSS CRC whose tumors had broadly similar immune cell infiltrate as determined by pathological analysis (KM score). We found increased frequencies of Treg cell populations in the colorectal tumor tissue, which positively correlated with the KM score. It has been established that increased immune infiltration is a positive prognostic factor in colorectal tumors. Our data showing a positive correlation between Treg infiltration and KM score therefore suggest that Treg cell infiltration may also correlate with improved outcomes. Given the prospective nature of our study, however, clinical outcomes were not yet available for our study cohort. Our findings are in broad agreement with others, which showed that increased Treg cell infiltration into colorectal tumors correlated with improved clinical outcomes (14, 15). This is in contrast to the case for the majority of other cancers, where Treg cell infiltration tends to be a negative prognostic indicator. Our findings and those of others showing Treg infiltration to be a positive prognostic factor seems counterintuitive, since it is generally assumed that Treg cells exert a pro-tumor role by suppressing antitumor immune responses. Treg cells have been shown to be enriched in parallel with effector T cells at inflamed peripheral sites within the body (25), most likely as an inherent mechanism to maintain homeostasis. However, for various reasons, Treg cells still fail to constrain inflammation at these sites of autoimmune inflammation (25). Colorectal tumors are known to be highly inflammatory relative to other tumor types (26). Thus, the enrichment of Treg cells within colorectal tumors may just be a reflection of the increased immune infiltration observed in colorectal tumors rather than an indication of a specific antitumor role for Treg cells.

Other studies did not find a positive correlation between good prognosis and Treg cell infiltration (16, 17). The reasons for these conflicting results are not clear. However, they may reflect some of the difficulties in identifying Treg cells by immunohistochemistry, since the use of sole Treg markers, such as CD25 or FoxP3, can lead to the false identification of activated effector T cells as Treg cells. In our study, using a flow cytometric approach, we were able to unequivocally identify Treg cells using a full panel of well accepted Treg markers. It is a matter of debate whether tumor infiltrating Treg cells act to inhibit antitumor effector T cells or other inflammatory responses, which may be proangiogenic, and thus protumorigenic (27).

We found that Tregs, which expressed the ectonucleotidase CD39, were enriched in colorectal tissue. CD39, which is normally expressed on a subset of human Treg cells in addition to other cells, acts to hydrolyze extracellular ATP to AMP, which is then further broken down to adenosine by CD73 (28). Adenosine binds to its cellular receptors, including the A2A receptor, on T cells where it exerts a suppressive effect. Thus, extracellular ATP, which is elevated in tumors, can be rapidly hydrolyzed by CD39 expressing Treg cells to mediate suppressive effects on T cells. It is likely therefore that the CD39^+^ Tregs, which we identified in colorectal tumors, will have a suppressive effect via adenosine. We also observed an increase in CD25^+^CD127^lo^ Treg cells, which were not all FoxP3^+^, although FoxP3^+^ Treg cells were included in this population. This was in agreement with a study that showed increased frequencies of FoxP3^−^ Treg cells in colorectal tumors, which were even more suppressive than FoxP3^+^ Treg cells and produced IL-10 and TGF-β (29). The function of Treg cells within colorectal tumors is of key importance, particularly if they are to be considered as targets for immunotherapy. We, therefore, sought possible correlations between the frequencies of tumor infiltrating Tregs and T cell cytokines. The frequencies of CD25^+^CD127^lo^ Treg cells correlated negatively with total T cell IFN-γ and IL-2, suggesting that these Treg cells may inhibit antitumor effector responses; however, there was no correlation between Treg cells and T cell IL-17 or TNF-α, both of which have been suggested to have pro-tumor activity (30, 31). Thus, it is possible that Treg cells are increased in colorectal tumors in proportion to the inflammatory infiltrate, where they may exert differentially suppressive effects on different subsets of T cells. The data suggest that Treg cells may be more effective at inhibiting T cells producing IFN-γ, such as Th1 and CTL, than those producing IL-17. Together with other immunosuppressive mechanisms within the tumor, this may help to tip the balance in favor of tumor progression.

Recent reports have described an aberrant subset of RORγt^+^/FoxP3^+^ IL-17-producing Treg cells (32). We therefore investigated whether the increased production of IL-17A by CD4 T cells within tumors was derived from FoxP3^+^ Treg cells. We saw that, although the majority of IL-17A in tumors came from FoxP3^−^ CD4 T cells, we also identified a population of IL-17^+^FoxP3^+^ CD4 T cells within tumors (data not shown). We also observed a positive correlation between IL-17 and IL-10 expression, although there was very little coexpression of these cytokines. The reason for the correlation between IL-17 and IL-10 expression is not clear; however, it has recently been shown that Th17 cells converted into IL-17^−^IL-10^+^ cells via a TGF-β-dependent mechanism (33). This may be a mechanism to downregulate inflammatory Th17 cells in the dynamic tumor environment.

In addition to Treg cells, the expression of PD-1 in tumors also provides important inhibitory signals in the tumor environment, where PD-1 expressing T cells can be rendered anergic by engaging their ligand PDL-1, which is expressed on tumor cells (19). We observed increased expression of PD-1 on both CD4 and CD8 T cells infiltrating colorectal tumors. PD-1 expression correlated negatively with T cell effector cytokines IFN-γ and IL-2, but not with IL-17. Increased expression of PD-1 on tumor infiltrating CD8 T cells correlated with an exhausted phenotype and reduced expression of IFN-γ and IL-2 (34). Thus, it appears that both Treg cells and PD-1 expressing cells may serve to inhibit effector T cells that produce IFN-γ and IL-2, but not IL-17, TNF-α, and IL-10. Since trials to test the efficacy of immune checkpoint inhibitors such as anti-PD-1 in CRC are ongoing, it will be crucial to understand the role of PD-1 in colorectal tumors. There appear to be some discrepancies in the literature regarding the levels of PD-1 expressed by tumor infiltrating lymphocytes, which are likely due to technical differences between studies. A study reporting that colorectal tumor infiltrating CD8^+^ T cells expressed little or no PD-1 employed a long (12-h) tissue digestion protocol (35), and this may have impacted on the phenotype of the recovered cells as determined by flow cytometry (36). In contrast, another study, which used a shorter digestion period, reported similar levels of PD-1 expression to those observed in our study (34). A further study investigating the expression of immune checkpoint inhibitors in MSI versus MSS CRC demonstrated PD-1 expression on both CD4 and CD8 T cells (37). Although the levels of PD-1, in the latter study, appear lower than those that we observed, the PD-1 data were expressed relative to that of T cells infiltrating normal adjacent colon tissue (PD-1 high). Thus, the absolute expression levels of PD-1 on tumor infiltrating T cells appear very similar to those that we observed (37). These discrepancies highlight the need for harmonization of methods and validation of PD-1 expression levels among different patient cohorts.

IFN-γ produced by both CD8 and CD4 T cells is considered to be a key antitumor effector. However, we did not observe any global differences in the expression of IFN-γ by CD4 or CD8 T cells in tumor versus adjacent uninvolved tissue. IL-17A, which was increased in tumor tissue, was the only cytokine with altered expression when compared with adjacent uninvolved tissue. IL-17A was produced primarily by CD4 T cells, which largely coproduced TNF-α. In contrast, only a small proportion of IL-17^+^ cells coexpressed IFN-γ. Th17 cells are known to be plastic and polyfunctional in terms of cytokine production and in the context of autoimmunity coproduction of IL-17, and IFN-γ is thought to be inflammatory and pathogenic, while in tumors such cells might be good antitumor effectors. However, the role of Th17 cells in cancer is still controversial, with both pro- and antitumor roles having been ascribed to IL-17. In CRC, the consensus appears to be that the expression of IL-17 may be a negative prognostic factor (4). Although IL-17 is an inflammatory cytokine that might be expected to have an antitumor effect, it is possible that its inflammatory effects rather serve to promote angiogenesis and promote tumor growth. Indeed, the angiogenic effects of IL-17 via the induction of VEGF have been documented (9). TNF-α also has angiogenic effects (30); thus, the Th17 cells that coproduce TNF-α, but not IFN-γ, which we have identified as being enriched in colorectal tumor tissue, may be particularly effective at inducing angiogenesis and thereby promoting tumor growth. Our data suggest that these cells may not be constrained by the regulatory networks that are prevalent in the tumor, including CD39^+^ Treg cells and PD-1 expressing T cells. This may have important implications for tumor therapy, and a fuller understanding of the role of Th17 cells and their regulation within colorectal tumors is warranted. Furthermore, TNF-α and IL-17A are thought to have synergistic tumor promoting properties when expressed together in CRC cells, whereby these cytokines stimulate glucose metabolism and growth factor production (38).

## Author Contributions

MD designed and performed flow cytometric analysis of tumor infiltrating and tissue T cell populations, analyzed data, contributed to the drafting of the manuscript, and approved the final version. CR and KS reviewed the pathology of all cases, performed the KM score analysis, contributed to the drafting of the manuscript, and approved the final version. BN, MT, RG, DW, PO, JH, and GD recruited and consented patients, provided clinical samples, compiled and interpreted clinical information, and approved final manuscript. JF and ER designed the experiments, drafted the manuscript, and approved the final version and are accountable for the accuracy and integrity of the work.

## Conflict of Interest Statement

The authors declare that the research was conducted in the absence of any commercial or financial relationships that could be construed as a potential conflict of interest.

## Abbreviations

CRC, colorectal cancer; CTLA-4, cytotoxic T lymphocyte antigen 4; DMSO, dimethyl sulfoxide; EMVI, extramural vascular invasion; FBS, fetal bovine serum; IL, interleukin; LVI, lymphovascular invasion; NI, neural invasion; PBMC, peripheral blood mononuclear cells; PBS, phosphate-buffered saline; PD-1, programed cell death 1; PENSTREP, penicillin streptomycin; PMA, phorbol myristate acetate; TNM, tumor node metastasis; Treg cell, regulatory T cell.

## References

[1] Pages F Kirilovsky A Mlecnik B Asslaber M Tosolini M Bindea G In situ cytotoxic and memory T cells predict outcome in patients with early-stage colorectal cancer. J Clin Oncol (2009) 27(35):5944–51. 10.1200/JCO.2008.19.6147 19858404

[2] Galon J Costes A Sanchez-Cabo F Kirilovsky A Mlecnik B Lagorce-Pages C Type, density, and location of immune cells within human colorectal tumors predict clinical outcome. Science (2006) 313(5795):1960–4. 10.1126/science.1129139 17008531

[3] Galon J Mlecnik B Bindea G Angell HK Berger A Lagorce C Towards the introduction of the ‘Immunoscore’ in the classification of malignant tumours. J Pathol (2014) 232(2):199–209. 10.1002/path.4287 24122236 PMC4255306

[4] Tosolini M Kirilovsky A Mlecnik B Fredriksen T Mauger S Bindea G Clinical impact of different classes of infiltrating T cytotoxic and helper cells (Th1, Th2, Treg, Th17) in patients with colorectal cancer. Cancer Res (2011) 71(4):1263–71. 10.1158/0008-5472.CAN-10-2907 21303976

[5] Muranski P Borman ZA Kerkar SP Klebanoff CA Ji Y Sanchez-Perez L Th17 cells are long lived and retain a stem cell-like molecular signature. Immunity (2011) 35(6):972–85. 10.1016/j.immuni.2011.09.019 22177921 PMC3246082

[6] Kryczek I Zhao E Liu Y Wang Y Vatan L Szeliga W Human TH17 cells are long-lived effector memory cells. Sci Transl Med (2011) 3(104):104ra0. 10.1126/scitranslmed.3002949 21998407 PMC3345568

[7] Martin-Orozco N Muranski P Chung Y Yang XO Yamazaki T Lu S T helper 17 cells promote cytotoxic T cell activation in tumor immunity. Immunity (2009) 31(5):787–98. 10.1016/j.immuni.2009.09.014 19879162 PMC2787786

[8] Charles KA Kulbe H Soper R Escorcio-Correia M Lawrence T Schultheis A The tumor-promoting actions of TNF-alpha involve TNFR1 and IL-17 in ovarian cancer in mice and humans. J Clin Invest (2009) 119(10):3011–23. 10.1172/JCI39065 19741298 PMC2752076

[9] Liu J Duan Y Cheng X Chen X Xie W Long H IL-17 is associated with poor prognosis and promotes angiogenesis via stimulating VEGF production of cancer cells in colorectal carcinoma. Biochem Biophys Res Commun (2011) 407(2):348–54. 10.1016/j.bbrc.2011.03.021 21396350

[10] Chung AS Wu X Zhuang G Ngu H Kasman I Zhang J An interleukin-17-mediated paracrine network promotes tumor resistance to anti-angiogenic therapy. Nat Med (2013) 19(9):1114–23. 10.1038/nm.3291 23913124

[11] Su X Ye J Hsueh EC Zhang Y Hoft DF Peng G. Tumor microenvironments direct the recruitment and expansion of human Th17 cells. J Immunol (2010) 184(3):1630–41. 10.4049/jimmunol.0902813 20026736

[12] Ling KL Pratap SE Bates GJ Singh B Mortensen NJ George BD Increased frequency of regulatory T cells in peripheral blood and tumour infiltrating lymphocytes in colorectal cancer patients. Cancer Immun (2007) 7:7. 17388261 PMC2935744

[13] Zhuo C Xu Y Ying M Li Q Huang L Li D FOXP3+ Tregs: heterogeneous phenotypes and conflicting impacts on survival outcomes in patients with colorectal cancer. Immunol Res (2015) 61(3):338–47. 10.1007/s12026-014-8616-y 25608795

[14] Frey DM Droeser RA Viehl CT Zlobec I Lugli A Zingg U High frequency of tumor-infiltrating FOXP3(+) regulatory T cells predicts improved survival in mismatch repair-proficient colorectal cancer patients. Int J Cancer (2010) 126(11):2635–43. 10.1002/ijc.24989 19856313

[15] Salama P Phillips M Grieu F Morris M Zeps N Joseph D Tumor-infiltrating FOXP3+ T regulatory cells show strong prognostic significance in colorectal cancer. J Clin Oncol (2009) 27(2):186–92. 10.1200/JCO.2008.18.7229 19064967

[16] Nosho K Baba Y Tanaka N Shima K Hayashi M Meyerhardt JA Tumour-infiltrating T-cell subsets, molecular changes in colorectal cancer, and prognosis: cohort study and literature review. J Pathol (2010) 222(4):350–66. 10.1002/path.2774 20927778 PMC3033700

[17] Betts G Jones E Junaid S El-Shanawany T Scurr M Mizen P Suppression of tumour-specific CD4(+) T cells by regulatory T cells is associated with progression of human colorectal cancer. Gut (2012) 61(8):1163–71. 10.1136/gutjnl-2011-300970 22207629 PMC3388728

[18] Ott PA Hodi FS Robert C. CTLA-4 and PD-1/PD-L1 Blockade: new immunotherapeutic modalities with durable clinical benefit in melanoma patients. Clin Cancer Res (2013) 19(19):5300–9. 10.1158/1078-0432.CCR-13-0143 24089443

[19] Ribas A Releasing the brakes on cancer immunotherapy. N Engl J Med (2015) 373(16):1490–2. 10.1056/NEJMp1510079 26348216

[20] Topalian SL Hodi FS Brahmer JR Gettinger SN Smith DC McDermott DF Safety, activity, and immune correlates of anti-PD-1 antibody in cancer. N Engl J Med (2012) 366(26):2443–54. 10.1056/NEJMoa1200690 22658127 PMC3544539

[21] Le DT Uram JN Wang H Bartlett BR Kemberling H Eyring AD PD-1 blockade in tumors with mismatch-repair deficiency. N Engl J Med (2015) 372(26):2509–20. 10.1056/NEJMoa1500596 26028255 PMC4481136

[22] Hanahan D Weinberg RA Hallmarks of cancer: the next generation. Cell (2011) 144(5):646–74. 10.1016/j.cell.2011.02.013 21376230

[23] Klintrup K Mäkinen JM Kauppila S Vare PO Melkko J Tuominen H Inflammation and prognosis in colorectal cancer. Eur J Cancer (2005) 41(17):2645–54. 10.1016/j.ejca.2005.07.017 16239109

[24] Vayrynen JP Sajanti SA Klintrup K Makela J Herzig KH Karttunen TJ Characteristics and significance of colorectal cancer associated lymphoid reaction. Int J Cancer (2014) 134(9):2126–35. 10.1002/ijc.28533 24154855

[25] Basdeo SA Moran B Cluxton D Canavan M McCormick J Connolly M Polyfunctional, pathogenic CD161+ Th17 lineage cells are resistant to regulatory T cell-mediated suppression in the context of autoimmunity. J Immunol (2015) 195(2):528–40. 10.4049/jimmunol.1402990 26062995

[26] Grivennikov SI Wang K Mucida D Stewart CA Schnabl B Jauch D Adenoma-linked barrier defects and microbial products drive IL-23/IL-17-mediated tumour growth. Nature (2012) 491(7423):254–8. 10.1038/nature11465 23034650 PMC3601659

[27] Whiteside TL. Regulatory T cell subsets in human cancer: are they regulating for or against tumor progression? Cancer Immunol Immunother (2014) 63(1):67–72. 10.1007/s00262-013-1490-y 24213679 PMC3888225

[28] Deaglio S Dwyer KM Gao W Friedman D Usheva A Erat A Adenosine generation catalyzed by CD39 and CD73 expressed on regulatory T cells mediates immune suppression. J Exp Med (2007) 204(6):1257–65. 10.1084/jem.20062512 17502665 PMC2118603

[29] Scurr M Ladell K Besneux M Christian A Hockey T Smart K Highly prevalent colorectal cancer-infiltrating LAP Foxp3 T cells exhibit more potent immunosuppressive activity than Foxp3 regulatory T cells. Mucosal Immunol (2013) 7(2):428–39. 10.1038/mi.2013.62 24064667 PMC3931584

[30] Leibovich SJ Polverini PJ Shepard HM Wiseman DM Shively V Nuseir N. Macrophage-induced angiogenesis is mediated by tumour necrosis factor-alpha. Nature (1987) 329(6140):630–2. 10.1038/329630a0 2443857

[31] Numasaki M Fukushi J Ono M Narula SK Zavodny PJ Kudo T Interleukin-17 promotes angiogenesis and tumor growth. Blood (2003) 101(7):2620–7. 10.1182/blood-2002-05-1461 12411307

[32] Blatner NR Mulcahy MF Dennis KL Scholtens D Bentrem DJ Phillips JD Expression of RORgammat marks a pathogenic regulatory T cell subset in human colon cancer. Sci Transl Med (2012) 4(164):164ra59 10.1126/scitranslmed.3004566 PMC3762575 23241743

[33] Gagliani N Vesely MC Iseppon A Brockmann L Xu H Palm NW Th17 cells transdifferentiate into regulatory T cells during resolution of inflammation. Nature (2015) 523(7559):221–5. 10.1038/nature14452 25924064 PMC4498984

[34] Wu X Zhang H Xing Q Cui J Li J Li Y PD-1(+) CD8(+) T cells are exhausted in tumours and functional in draining lymph nodes of colorectal cancer patients. Br J Cancer (2014) 111(7):1391–9. 10.1038/bjc.2014.416 25093496 PMC4183848

[35] Droeser RA Hirt C Viehl CT Frey DM Nebiker C Huber X Clinical impact of programmed cell death ligand 1 expression in colorectal cancer. Eur J Cancer (2013) 49(9):2233–42. 10.1016/j.ejca.2013.02.015 23478000

[36] Abuzakouk M Feighery C O’Farrelly C. Collagenase and dispase enzymes disrupt lymphocyte surface molecules. J Immunol Methods (1996) 194(2):211–6. 10.1016/0022-1759(96)00038-5 8765174

[37] Llosa NJ Cruise M Tam A Wicks EC Hechenbleikner EM Taube JM The vigorous immune microenvironment of microsatellite instable colon cancer is balanced by multiple counter-inhibitory checkpoints. Cancer Discov (2015) 5(1):43–51. 10.1158/2159-8290.CD-14-0863 25358689 PMC4293246

[38] Straus DS TNFalpha and IL-17 cooperatively stimulate glucose metabolism and growth factor production in human colorectal cancer cells. Mol Cancer (2013) 12:78 10.1186/1476-4598-12-78 23866118 PMC3725176

